# *In Vitro* Antibacterial and Antifungal Activity of *Lavandula* × *intermedia* Emeric ex Loisel. ‘Budrovka’

**DOI:** 10.3390/molecules16054241

**Published:** 2011-05-23

**Authors:** Biljana Blazekovic, Gordana Stanic, Stjepan Pepeljnjak, Sanda Vladimir-Knezevic

**Affiliations:** 1 Department of Pharmacognosy, Faculty of Pharmacy and Biochemistry, University of Zagreb, Marulićev trg 20, Zagreb 10000, Croatia; E-Mails: bblazekovic@pharma.hr (B.B.); gordanas@pharma.hr (G.S.); 2 Department of Microbiology, Faculty of Pharmacy and Biochemistry, University of Zagreb, Schrottova 39, Zagreb 10000, Croatia; E-Mail: spep33@yahoo.com (S.P.)

**Keywords:** *Lavandula* × *intermedia* ‘Budrovka’, ethanolic extracts, antibacterial, antifungal

## Abstract

This study aimed to evaluate the *in vitro* antibacterial and antifungal activities of *Lavandula* × *intermedia* Emeric ex Loisel. ‘Budrovka’, an indigenous Croatian cultivar of lavandin. For that purpose the activity of ethanolic extracts of flowers, inflorescence stalks and leaves against thirty one strains of bacteria, yeasts, dermatophytes and moulds were studied using both the agar well diffusion and broth dilution assays. Among the investigated extracts found to be effective against a broad spectrum of microorganisms, the flower extract was considered to be the most potent one. Linalool and rosmarinic acid, as the most abundant constituents found, are very likely major contributors to the observed antimicrobial effects. The results suggest that flowers of lavandin ‘Budrovka’ could serve as a rich source of natural terpene and polyphenol antimicrobial agents.

## 1. Introduction

Plant-based remedies have been part of traditional health care in most parts of the World for thousands of years and there is increasing scientific interest in plants as a source of novel agents to fight infectious diseases. Since the discovery and exploitation of antibiotic agents in the 20th century, the targeted selective toxicity of such agents has ensured their widespread and largely effective use to combat infection; however it has paradoxically resulted in the emergence and dissemination of multi-drug resistant pathogens [1,2]. The quest for a solution to the global problem of antibiotic resistance has often focused on finding new antimicrobial compounds from a variety of natural sources. Plants produce numerous small-molecule compounds which exhibit various biological properties that can be utilized in the treatment of various human diseases. Useful antimicrobial phytochemicals can be divided into several categories such as terpenoids, flavonoids, tannins, saponins, alkaloids, and other compounds reported to have antimicrobial properties [3,4]. From these points of view, it is obvious that natural products, either in the form of pure compounds or as standardized plant extracts, provide unlimited opportunities to develop a wide range of new drugs. Besides the health aspect, there is an increasing tendency for the use of plant-derived antimicrobial agents as natural preservatives.

The Lamiaceae family includes numerous medicinal herbs with pronounced therapeutic properties such as *Lavandula* species, aromatic plants mainly distributed in the Mediterranean area. Well-known since ancient times, the medicinal uses of lavender inflorescence are based on its sedative, cholagogue, spasmolytic, carminative and antiseptic properties [5]. *Lavandula* species are also grown for their wide range of uses in perfumery, cosmetics and food processing. Previous phytochemical studies have indicated that *Lavandula* species contain essential oil, triterpenes, coumarins, hydroxycinnamic acids and flavonoids [6]. *Lavandula* × *intermedia* Emeric ex Loisel. ‘Budrovka’ is a Croatian lavandin cultivar, widely cultivated for the production of essential oil. Our recent findings highlighted this indigenous hybrid as a rich source of polyphenolic compounds found to be responsible for its antioxidant activity [7]. Although some previous studies mention the antimicrobial action of several essential oils isolated from related *Lavandula* species, non-volatile extracts were generally very poorly studied [8,9,10]. Moreover, there is no any literature data concerning any potential antibacterial and antifungal effects of lavandin ‘Budrovka’.

In the search for new antimicrobial resources, the present work focuses on investigating the *in vitro* activities of *L.* × *intermedia* ‘Budrovka’ against a broad spectrum of human pathogenic bacteria and fungi, as well as microorganisms causing the spoilage of food, pharmaceutical and cosmetic products.

## 2. Results and Discussion

### 2.1. Phytochemical Analyses

The antimicrobial activity of plant extracts depends strongly on the type and amount of active principles. Their contents and composition vary from plant to plant species and even in different parts of the same species [4]. Therefore, qualitative and quantitave analyses of constituents present are very important in both the plant material and related extracts examined.

In the first step, the contents of essential oil and individual polyphenolic subclasses were determined in flowers, inflorescence stalks and leaves of *L.* × *intermedia* ‘Budrovka’. As can be seen from the Table 1, flowers contained a markedly high amount of essential oil (6.80%), while leaves contained much less (0.40%) and inflorescence stalks did not provide any detectable quantity of essential oil using the hydrodistillation method. The contents of tannins, phenolic acids and flavonoids were determined by different colorimetric methods, and ranged widely from 0.06% to 4.49%, with statistically significant differences between the plant organs (p < 0.01). The majority were found to be located in the leaves and flowers, which contained 5.82% and 5.20% of the total polyphenolic compounds, respectively, while their content in inflorescence stalks was only 1.80%. Phenolic acids were the most representative subclass of polyphenols in all plant organs examined (1.29–4.49%). The results of quantitative analyses therefore indicated that the flowers of lavandin ‘Budrovka’ are much richer in essential oil than other parts of the plant, and also contain a high amount of polyphenols, similar to that found in leaves.

**Figure 1:**
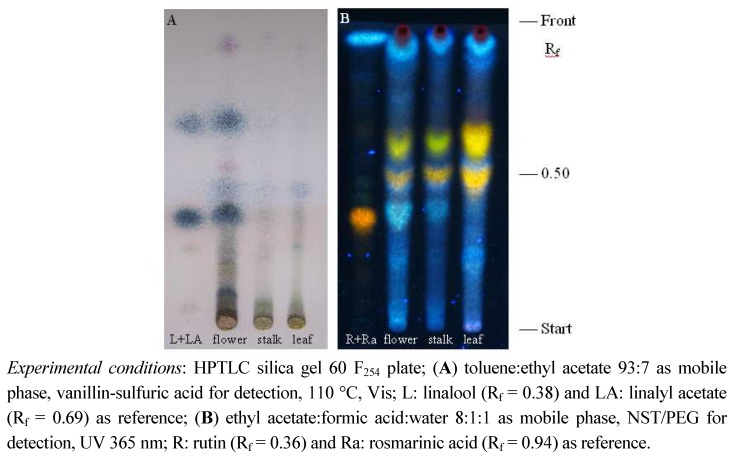
HPTLC chromatogram of terpenic (**A**) and polyphenolic (**B**) constituents of *Lavandula* × *intermedia* ‘Budrovka’ flower, inflorescence stalk and leaf extracts.

Since the biological activities of aromatic plants are considerably dependant on volatiles content, liquid lavandin ‘Budrovka’ extracts were prepared, thus minimizing the undesirable heating and evaporation so their effectiveness would not be susceptible to impairment. The ethanolic extracts in question were screened by HPTLC for the presence of terpenic compounds and individual polyphenolic subclasses previously recognized to have antimicrobial potential [11,12]. As can be seen in the Figure 1A, the chromatogram obtained with the flower ethanolic extract shows two intensive blue spots corresponding to linalool and linalyl acetate (R_f_ values 0.38 and 0.69, respectively), which indicate great amounts of these compounds. In contrary, only traces of terpenes were detected in ethanolic extracts of inflorescence stalk and leaf. The presence of flavonoids and phenolic acid corresponding to yellow and blue spots, respectively, was proved in all extracts studied (Figure 1B). The fluorescence intensity of related spots shows that the extract prepared from leaves contains higher amounts of flavonoids than the flower and inflorescence stalk extracts. Moreover, rosmarinic acid is detected as one of the most abundant polyphenolic constituents in all extracts tested.

### 2.2. Antibacterial and Antifungal Effects

In this study two different assays were applied to examine the activity of liquid extracts of *L.* × *intermedia* ‘Budrovka’ on a large number of microorganisms evaluating the antimicrobial property of this indigenous Croatian cultivar of lavandin, with the assumption of the great potential for the cultivation and practical applications of this plant.

Antibacterial and antifungal activities of different lavandin ‘Budrovka’ ethanolic extracts were assessed according to the inhibition zones (IZ) as well as the minimum inhibitory concentration (MIC) values and minimum bactericidal/fungicidal concentrations (MBC/MFC). As shown in the Table 2, flower extract was found to be effective against all tested bacteria (IZ: 8–22 mm), while several Gram-negative bacterial strains showed little or no susceptibility to leaf and stalk extracts. Antibacterial activity of the extracts applied using diffusion method decreased in order of flower > leaf > inflorescence stalk. The largest inhibition zones were found in the activity of all extracts against *Streptococcus pyogenes* (14–22 mm). MIC and MBC values were determined using the dilution method, and the results are expressed in volume percentages (Table 2). The extracts showed activity against all tested bacteria, and the strongest antibacterial effect was observed for the flower extract (MIC: 0.1–1.5%; MBC: 0.2–2.5%), followed by the leaf extract (MIC: 1.25–10.0%; MBC: 2.5–12.5%), while the extract of inflorescence stalks proved to be the least effective (MIC: 2.5–12.5%; MBC: 5.0–15.0%). No obvious difference in susceptibility was found among Gram-positive and Gram-negative bacteria tested. Among them, the most sensitive to the flower extract was *Pseudomonas aeruginosa*, which tends to develop resistance to commonly used antibiotics.

Table 3 presents the results of screening of antifungal activity using both diffusion and dilution methods. Like the reference antimycotic, flower extract was effective against all tested yeasts and moulds (IZ: 10–21 mm) and dermatophytes (IZ: 30–33 mm), while the leaf and stem extracts did not generate the inhibition zone for all tested strains. Furthermore, the diameters of inhibition zone formed were substantially smaller than those for the extract of flowers. A strong antifungal effect of the flower extracts was also confirmed using the dilution method (MIC: 0.05–2.5%; MFC: 0.1–5.0%). However, the MIC/MFC values for leaf and stalk extracts were significant higher (2.0–40.0%). *Candida krusei* was one of the most sensitive fungi using dilution method, while all tested moulds were found to be the most resistant strains. Generally, dermatophytes proved to have a great susceptibility to the fluid ethanolic extracts of *L.* × *intermedia* ‘Budrovka’ in the both assays applied.

The presented results demonstrate that all investigated extracts possess the ability to inhibit either bacterial or fungal growth. Though the activities against the tested microorganisms were of varying intensity, the results clearly proved that ethanolic extracts of *L.* × *intermedia* ‘Budrovka’ have antimicrobial capacities and act against a broad spectrum of bacteria, yeasts, moulds and dermatophytes, with flower extract proving to be the most potent one. Our findings suggest that the antimicrobial effects of the extracts examined could be associated with their phenolic constituents. In addition, linalool and linalyl acetate observed in a relatively huge amount in the flower extract have been already considered as a strong antimicrobial agents, notably linalool. Dorman and Deans [13] showed that linalool exhibit potent activity against most of the twenty five bacterial strains tested, causing zones of growth inhibition in the range of 8–28 mm. Similar antibacterial properties of this monoterpene alcohol (IZ: 8–20 mm) were reported by Soković *et al.* [14], with MICs 4.0–7.0 μg/mL, while for the linalyl acetate significantly lower activity was noticed (IZ: 6–12 mm; MICs 7.0–10.0 μg/mL). Since the flower extract exhibited much stronger activity than extract prepared from leaves containing the high level of polyphenols, these monoterpenic compounds are most likely to have the greatest impact on the antimicrobial capacity. Besides, it is important to accentuate that plant extracts can affect not only a single target but various targets, where the different active components collaborate in a synergistic-agonistic manner. The use of extracts as antimicrobial agents shows a low risk of increasing resistance to their action, because they are complex mixtures, making microbial adaptability very difficult [15]. Our results also indicated that flowers of *L.* × *intermedia* ‘Budrovka’ could serve as a rich source of antimicrobial agents which could be effective against bacteria having ability to develop antibiotic resistance. In addition to the proven activity against a broad spectrum of pathogenic bacteria, a strong antifungal activity of the flower extract was found against dermatophytes and *Candida* species, which are both causal agents of serious skin and mucous membrane infections that require long-term treatment. In this connection, our study suggests the possibility of external use of *L.* × *intermedia* ‘Budrovka’ flower extract, and encourages future *in vivo* research too.

Microbial contamination of food as well as pharmaceuticals and cosmetics is significant both from public health and economic viewpoints. Utilization of plant extracts as an alternative to synthetic antimicrobial and antioxidant chemicals is an increasing trend since natural additives are considered to be safer and less toxic [16,17]. The flower extracts evaluated in this study showed inhibitory effect against all foodborne and spoiling microbial strains tested.

Additionally, our recently published study [7] reported the strong antioxidant activity of the flower ethanolic extract, which was mainly due to the presence of polyphenolic compounds. These findings, considered together, make *L.* × *intermedia* ‘Budrovka’ flowers a good candidate for further research with a view to finding a safe and effective natural preservative.

## 3. Experimental

### 3.1. Plant Material

Aerial parts of *Lavandula* × *intermedia* Emeric ex Loisel. ‘Budrovka’ were collected at full flowering stage from plants cultivated at a farm in the village of Dragovanščak, near the city of Jastrebarsko (Central Croatia, 45°70’ N, 15°55’ E). Plant material was air-dried and leaves, flowers and inflorescence stalks were separated. The plant sample was identified at the Department of Pharmacognosy, Faculty of Pharmacy and Biochemistry, University of Zagreb, Croatia, where a voucher specimen has been deposited (Number FBF-FGN/BB 101).

### 3.2. Preparation of Plant Extracts

Liquid extracts of flowers, inflorescence stalks and leaves from *L.* × *intermedia* ‘Budrovka’ were prepared by the percolation procedure according to European Pharmacopoeia [18]. Briefly, the plant sample (30 g) reduced to the suitable size was mixed thoroughly with a portion of the 80% ethanol (15 mL) and allowed to stand for about 2 h. The mixture was then transferred to a percolator, and a sufficient amount of the solvent was added to cover the entire solid mass which was than left to macerate for 24 h. Afterwards, the percolation was performed at a rate of 1–3 mL/min, gradually adding 80% ethanol until plant material was exhausted. The percolated liquid was concentrated to the volume of 30 mL under reduced pressure at a temperature below 50 °C, so that each mL of the final extract contained the constituents that could be extracted from 1 g of the dried plant material.

### 3.3. Phytochemical Analyses

#### 3.3.1. Determination of essential oil

The contents of essential oil in flower, inflorescence stalk and leaf of lavandin ‘Budrovka’ were determined by hydrodistillation method, according to the procedure described in the European Pharmacopoeia [18]. In short, the dried plant material (20.0 g) was placed in the flask of a Clevenger-type apparatus and extracted with water steam (500 mL) for 2 h. The distillate was collected in a graduated tube using xylene to take up the essential oil. Ten min after the heating was stopped, the volume of liquid collected was read and the blank xylene value, determined in a parallel distillation in the absence of the vegetable drug, was subtracted. Yield percentage was expressed as volume (mL) of essential oil per 100 g of plant dry matter. All determinations were performed in triplicate. 

#### 3.3.2. Determination of total tannins

Determination of total tannin contents was performed following the method described in European Pharmacopoeia [18]. Briefly, the powdered plant sample (0.5 g) was boiled for 30 min in a water bath with water (150 mL), then the filtrate was made up to 250 mL with water and the obtained solution served as stock solution. An aliquot of stock solution was mixed with Folin-Ciocalteu’s phenol reagent and sodium carbonate solution. After 30 min, the absorbance was read at 760 nm (A_1_), and the quantification of total phenols was done with respect to the standard calibration curve of pyrogallol (6.25–50.00 mg). For the determination of tannins content, stock solution was vigorously shaken with hide powder for 60 min. Since the hide powder adsorbed tannins, phenols unadsorbed on hide powder were measured in filtrate, after addition of Folin-Ciocalteu’s phenol reagent in a sodium carbonate medium (A_2_). The percentage content of tannins, expressed as pyrogallol, was calculated from the following equation: *(%) = 3.125 × (A_1_ − A_2_) / (A_3_ × m)*, where *A_3_* is the absorbance of the test solution containing 0.05 g of pyrogallol, and *m* the mass of the extract (g).

#### 3.3.3. Determination of total phenolic acids

Determination of hydroxycinnamic acid derivatives was performed according to procedure described in European Pharmacopoeia [18]. Briefly, the powdered plant material (0.20 g) was extracted with 50% ethanol (80 mL) under a reflux condenser in a boiling water bath for 30 min. The cooled extract was filtered, the filter rinsed with ethanol, and then the combined filtrate and rinsing solution was diluted to 100.0 mL with 50% ethanol. An aliquot of the extract (1.0 mL) was mixed with 0.5 M hydrochloric acid (2 mL), Arnow reagent (10% aqueous solution of sodium nitrite and sodium molybdate, 2 mL), and 8.5% sodium hydroxide (2 mL) and diluted to 10.0 mL with water. The absorbance of the test solution was measured immediately at 505 nm against sample blank. The percent of total hydroxycinnamic acid content was calculated and expressed as rosmarinic acid, according to the following expression: *(%) = A × 2.5 / m*, where *A* is the absorbance of the test solution at 505 nm and *m* is the mass of the sample, in grams. Analysis of each sample was performed in triplicate.

#### 3.3.4. Determination of total flavonoids

The total flavonoid contents in *L.* × *intermedia* ‘Budrovka’ flower, inflorescence stalk and leaf, respectively, were determined using the spectrophotometric method of Christ and Müller [19]. Each powdered plant sample (0.20 g) was mixed with acetone (20 mL), 25% hydrochloric acid (2 mL) and 0.5% hexamethylenetetramine solution (1 mL) and heated under reflux in a water bath for 30 min. The extract was filtered and re-extracted twice with acetone (20 mL) for 10 min. Filtrates were combined and made up to 100.0 mL with acetone. An aliquot of the acetone extract (20 mL) was mixed with water (20 mL) and then extracted with three portions of ethyl acetate (15 mL each). The combined ethyl acetate layers were washed twice with water then filtered and diluted to 50.0 mL. To this solution (10.0 mL) 0.5% solution of sodium citrate (0.5 mL) and 2% aluminium chloride solution (in 5% methanolic solution of acetic acid, 2 mL) was added and then diluted to 25.0 mL with 5% methanolic solution of acetic acid. The mixture was allowed to stand for 45 min and the absorbance was measured at 425 nm. A sample solution prepared in the same manner but without addition of aluminium chloride solution served as a blank. All determinations were performed in triplicate. The percentage content of flavonoids, expressed as quercetin, was calculated from the equation: *(%) = A × 0.772 / m*, where *A* is the absorbance of the test solution at 425 nm and *m* is the mass of the sample, in grams.

#### 3.3.5. HPTLC analyses

High performance thin-layer chromatographic (HPTLC) analyses of different secondary plant metabolites was performed on precoated silica gel 60 F_254_ HPTLC plates (Merck, Germany). Aliquots (10 μL) of fluid ethanolic extracts and 0.5% ethanolic solution of reference substances were spotted on to the plates which were then developed in vertical glass chambers previously saturated with the mobile phases: toluene:ethyl acetate (93:7, v/v), ethyl acetate:formic acid:water (8:1:1, v/v/v), diisopropyl ether-acetone:water:formic acid (50:30:10:10, v/v/v/v) [20,21,22] for analysis of terpenes, flavonoids and phenolic acids, respectively. After development plates were dried in a stream of air for a few minutes. For visualisation of terpenes in visible light, plates were sprayed with 10% ethanolic vanillin-sulfuric acid reagent and heated at 110 °C for 5 min. Flavonoids and phenolic acids were detected under UV light at 365 nm after spraying them with natural products-polyethylene glycol reagent (1% methanolic solution of 2-aminoethyl diphenylborinate and 5% ethanolic solution of PEG 4000, NST/PEG).

### 3.4. Antimicrobial Assays

#### 3.4.1. Test microorganisms and inocula preparation

A total of thirty-one tested microbial cultures belonging Gram-positive and Gram-negative bacteria, yeasts and dermatophytes are listed below in Table 2 and Table 3. They were provided by the Department of Microbiology, Faculty of Pharmacy and Biochemistry, University of Zagreb, Croatia. Microorganisms used in this study included the strains from their own collection as well as reference strains of American Type Culture Collection (ATCC) and National Collection of Type Cultures (NCTC).

All tested bacterial and fungal strains were maintained on an agar slant and stored at 4 °C. Inocula were prepared with fresh cultures by suspending the microorganisms in sterile saline and adjusting the density to 0.5 Mcfarland standard (10^8^ CFU/mL).

#### 3.4.2. Diffusion method

Antimicrobial activity testing of the *L.* × *intermedia* ‘Budrovka’ liquid extracts was carried out using the agar well diffusion method [18]. Briefly, culture medium was inoculated with the given microorganism by spreading the microbial suspension (1 mL) in the media, Mueller-Hinton agar for bacteria and Sabouraud dextrose agar for fungi, respectively. Wells were punched in the agar using a sterile metal cylinder (6 mm diameter) and the extract (50 µL) was placed into them. The plates were left 1 h at 4 °C to allow the diffusion of the extract and then incubated under aerobic conditions at 37 °C for bacteria and 28 °C for fungi. After an incubation period of 24 h for bacteria and 2–5 days for fungi, the diameters of the inhibition zones of microbial growth around the samples were measured in millimeters. Ethanol (80%) served as a negative control in all plates, while oxytetracycline (50 μg/mL) and nystatin (5 mg/mL) (Sigma) were used as positive reference standards to determine the sensitivity of each bacterial and fungal strains tested, respectively. All the tests were performed in triplicate.

#### 3.4.3. Dilution method

To determine the minimum inhibitory concentration (MIC) and minimum bactericidal/fungicidal concentration (MBC/MFC), the broth dilution method was applied [23,24]. Serial dilutions of the studied extracts were made in the liquid nutrient medium, Mueller-Hinton broth for bacteria and Sabouraud broth for fungi. Following the inoculation, the test tubes were incubated at 37 °C for 24 h (bacteria) and at 28 °C for 2–5 days (fungi). The MIC, which was taken as the lowest concentration of the extract that allows no more than 20% growth of the microorganism, and MBC/MFC as the lowest concentration of extract resulting in no bacterial/fungal growth were determined by subculturing ca. 10 μL of each test dilutions on the surface of an appropriate agar plate.

### 3.5. Statistical Analysis

Experiments were carried out in triplicate, and the results are expressed as mean ± standard deviation (SD). Differences were estimated by Student’s t-test and the values p < 0.05 were considered statistically significant.

## 4. Conclusions

The results presented in this paper can be considered the first report on the antimicrobial activity of *L.* × *intermedia* ‘Budrovka’, contributing also in a great extent to the current poor knowledge on the effectiveness of non-volatile extracts of *Lavandula* taxa against microorganisms in general. The performed studies demonstrated that all of the extracts prepared from the different plant parts, flower, inflorescence stalk and leaf, respectively, possess the ability to inhibit either bacterial or fungal growth although their activity varied greatly. Flower extract of *L.* × *intermedia* ‘Budrovka’ was found to be the most potent one, showing the capacity to act toward a broad spectrum of bacteria, yeasts, moulds and dermatophytes. Hence, lavandin ‘Budrovka’ flowers could be used or as an easy accessible source of natural antimicrobial agents for medicinal purposes or to meet the needs of food, pharmaceutical and cosmetic industry. Although our findings indicated that the monoterpenes linalool and linalylacetate, accompanied by polyphenolic constituents, are most probably the constitutents responsible for the strong antibacterial and antifungal effects of the flower extract, further research should aim to isolate the active compounds and to investigate their structure and effectiveness in greater detail.

## Figures and Tables

**Table 1:** Contents of bioactive constituents of different parts of *Lavandula* × *intermedia* ‘Budrovka’.

Bioactive constituents	Content (%)		
	Flower	Inflorescence stalk	Leaf
Essential oil	6.80 ± 0.20	nd	0.40 ± 0.05
Tannins	1.04 ± 0.02	0.41 ± 0.02	1.17 ± 0.03
Phenolic acids	4.10 ± 0.12	1.29 ± 0.07	4.49 ± 0.16
Flavonoids	0.06 ± 0.01	0.10 ± 0.01	0.16 ± 0.01

The contents of essential oil were determined by hydrodistillation method (%, V/m), while the amounts of various polyphenols were determined spectrophotometrically (%, m/m). Each value represents mean ± SD (n = 3); nd: not detectable using the hydrodistillation method.

**Table 2:** Antibacterial activity of *Lavandula* × *intermedia* ‘Budrovka’ ethanolic extracts.

Microorganism	Inhibition zone [mm]				MIC (MBC) [%, V/V]		
	Flower	Stalk	Leaf	OTC	Flower	Stalk	Leaf
*Bacillus cereus* ATCC 11778	18 ± 2	9 ± 1	13 ± 1	28 ± 1	0.25 (0.5)	5.0 (7.5)	2.5 (5.0)
*Bacillus pumilus* NCTC 8241	13 ± 0	9 ± 0	11 ± 1	25 ± 2	0.5 (0.75)	5.0 (7.5)	2.5 (5.0)
*Bacillus subtilis* NCTC 8236	13 ± 1	10 ± 0	12 ± 0	28 ± 2	0.2 (0.25)	2.5 (5.0)	1.25 (2.5)
*Enterococcus faecalis* ATCC 19433	19 ± 1	10 ± 1	9 ± 0	15 ± 2	1.25 (2.5)	12.5 (15.0)	10.0 (12.5)
*Escherichia coli* ATCC 25922	16 ± 2	8 ± 0	10 ± 0	na	0.5 (1.0)	10.0 (12.5)	7.5 (10.0)
*Klebsiella oxytoca*	11 ± 1	na	8 ± 0	20 ± 0	1.25 (2.5)	10.0 (12.5)	7.5 (10.0)
*Klebsiella pneumoniae* ATCC 10031	10 ± 0	na	na	16 ± 0	1.25 (2.5)	10.0 (12.5)	7.5 (10.0)
*Kocuria rhizophila* ATCC 9341	16 ± 1	na	9 ± 1	25 ± 2	1.5 (2.0)	12.5 (15.0)	7.5 (10.0)
*Listeria monocytogenes* ATCC 7644	15 ± 2	11 ± 1	13 ± 1	27 ± 1	0.5 (1.0)	7.5 (10.0)	5.0 (7.5)
*Proteus mirabilis*	8 ± 0	na	na	na	1.0 (1.5)	10.0 (12.5)	7.5 (10.0)
*Pseudomonas aeruginosa* ATCC 27853	17 ± 1	10 ± 0	12 ± 1	11 ± 2	0.1 (0.2)	5.0 (7.5)	2.0 (2.5)
*Salmonella enteritidis* ATCC 13076	12 ± 1	na	8 ± 0	19 ± 1	1.0 (1.5)	7.5 (10.0)	5.0 (7.5)
*Staphylococcus aureus* ATCC 25923	16 ± 2	10 ± 1	11 ± 2	21 ± 1	0.75 (1.5)	10.0 (12.5)	7.5 (10.0)
*Streptococcus pyogenes* ATCC 12204	22 ± 2	14 ± 1	17 ± 2	33 ± 2	0.25 (0.5)	5.0 (7.5)	2.5 (5.0)
*Yersinia enterocolitica*	16 ± 2	na	9 ± 0	na	1.0 (2.0)	10.0 (12.5)	7.5 (10.0)

The results of diffusion method are presented as diameters of inhibition zones in mm. Each value represents mean ± SD (n = 3). Minimal inhibitory concentrations (MIC) and minimal bactericidal concentrations (MBC) of fluid extract were determined by broth dilution assay and results are expressed as volume percentage (%, V/V). Oxytetracycline (OTC) was used as positive control; na: no activity.

**Table 3:** Antifungal activity of *Lavandula* × *intermedia* ‘Budrovka’ ethanolic extracts.

Microorganism	Inhibition zone [mm]				MIC (MFC) [%, V/V]		
	Flower	Stalk	Leaf	Nystatin	Flower	Stalk	Leaf
*Blastoschizomyces capitatus*	11 ± 1	na	9 ± 1	9 ± 0	0.75 (1.0)	17.5 (20.0)	10.0 (12.5)
*Candida albicans* ATCC 10231	13 ± 2	na	na	20 ± 1	0.5 (1.0)	12.5 (15.0)	10.0 (15.0)
*Candida glabrata*	10 ± 1	na	na	20 ± 1	0.25 (0.5)	20.0 (22.5)	15.0 (17.5)
*Candida krusei*	11 ± 1	8 ± 0	8 ± 0	11 ± 1	0.05 (0.1)	7.5 (10.0)	5.0 (7.5)
*Candida tropicalis*	21 ± 1	8 ± 0	10 ± 0	24 ± 2	0.5 (0.75)	10.0 (12.5)	7.5 (10.0)
*Cryptococcus neoformans*	13 ± 1	8 ± 0	8 ± 0	21 ± 1	0.15 (0.25)	10.0 (12.5)	7.5 (10.0)
*Hansenula anomala*	12 ± 1	na	8 ± 0	19 ± 2	1.5 (2.0)	7.5 (10.0)	7.5 (10.0)
*Microsporum gypseum*	30 ± 0	8 ± 0	13 ± 1	8 ± 0	1.0 (2.0)	5.0 (7.5)	2.5 (5.0)
*Microsporum canis*	30 ± 2	8 ± 0	15 ± 1	9 ± 1	1.0 (2.0)	5.0 (7.5)	2.5 (5.0)
*Trichophyton rubrum*	33 ± 1	10 ± 1	15 ± 2	8 ± 1	0.25 (0.5)	5.0 (7.5)	2.0 (4.0)
*Trichophyton mentagrophytes*	31 ± 2	9 ± 1	14 ± 1	8 ± 1	1.5 (2.0)	5.0 (7.5)	2.0 (4.0)
*Aspergillus niger* ATCC 16404	10 ± 1	na	8 ± 1	17 ± 1	2.5 (5.0)	37.5 (40.0)	30.0 (35.0)
*Aspergillus fumigatus*	20 ± 2	na	na	17 ± 2	2.5 (5.0)	37.5 (40.0)	30.0 (35.0)
*Fusarium oxysporum*	12 ± 1	na	8 ± 1	20 ± 1	2.0 (4.0)	15.0 (20.0)	15.0 (17.5)
*Penicillium citrinum*	10 ± 1	na	8 ± 1	11 ± 1	2.5 (5.0)	22.5 (25.0)	20.0 (22.5)
*Trichoderma viride*	18 ± 2	na	na	20 ± 1	2.0 (4.0)	15.0 (20.0)	12.5 (15.0)

The results of diffusion method are presented as diameters of inhibition zones in mm. Each value represents mean ± SD (n = 3). Minimal inhibitory concentrations (MIC) and minimal fungicidal concentrations (MBC) of fluid extract were determined by broth dilution assay and results are expressed as volume percentage (%, V/V). Nystatin was used as positive control; na: no activity.
